# New York Odontological Society

**Published:** 1893-09

**Authors:** 

**Affiliations:** New York Odontological Society


### NEW YORK ODONTOLOGICAL SOCIETY.

A REGULAR meeting of the New York Odontological Society was held on Tuesday evening, June 20, 1893, at the New York Academy of Medicine, No. 17 West Forty-third Street, New York City, with the President, Dr. Woodward, in the chair.

In the absence of the Secretary, Dr. Ives read the minutes of the last meeting, which were approved.

The order of business was changed so as to allow the paper to come first. Dr. Mayr, of Springfield, therefore read his paper entitled,—

### DOES THE QUALITY OF AMALGAM DEPEND ON ATOMIC FORMULÆ?

If there existed amalgams anywhere in nature similar to those used by the dental profession, we might learn a lesson from them. The following amalgams have been found:

Silver, thirty-four per cent.; mercury, sixty-six per cent., somewhat according to the formula $AgHg_2$. Another amalgam, silver, twenty-six per cent.; mercury, seventy-three per cent.; $AgHg_3$. They are crystallized, dodecahedral, and very white and brittle.

From South America the following compounds have been observed:

$Hg_{56}Ag_{44}$, color, white; soft.

$Hg_{53}Ag_{47}$, color of lead; fairly hard.

$Hg_{45}Ag_{55}$, hard; color, steel.

$Hg_{47}Ag_{53}$, very hard; dark.

$Hg_{36}Ag_{64}$.

$Hg_{13}Ag_{87}$, very white, like silver; almost the same hardness.

Two gold-mercury amalgams, very soft and brittle, have been observed: one, $Au_{39}Hg_{61}$; another, $Au_{42}Hg_{58}$; both found in California.

A gold, silver, and mercury amalgam, containing $Hg_{57}Au_{38}Ag_5$, has also been observed. The latter would be entirely worthless, because it crumbles even under the fingers.

From these mineralogical occurrences it is plain that gold does not improve the quality of an amalgam. It may improve the setting, salability, and other side issues, but not the intrinsic quality.

I fail to see why there exists such a general superstition about gold; because it is the standard of money does not prove that it is intrinsically of greater value. If gold was not used for a standard of money it would be a great drug on the market, and would rank in intrinsic value about with copper, compared with which it would have the great drawback of a much greater specific gravity. It is with gold as with man: those with the highest newspaper reputations are not by any means intrinsically the best men; quite the contrary. As an example I may quote Virchow, the famous German pathologist, who entered politics, and has not done anything of intrinsic value since the newspapers began to notice and "boom" him. His "Cellular Pathology" he published when a most unknown young teacher; he has become a scientific nonentity since he became a candidate for congresses and legislatures, but a shining public light.

It may be said that a man while obscure constitutes an average of his own; as soon as he becomes public he becomes the average of his constituency, which in almost all cases is away below his own.

This to emphasize my point about gold in amalgams.

No natural amalgam exists which contains silver, *tin*, and mercury; but it may be said that all dental amalgams contain more or less tin. Now, from a theoretical stand-point, tin is considered —whether it is so or not I do not know—a quadrivalent element,—that is, a substance belonging to the carbon group, to which belong carbon, silicon, titanium, germanium, tin, sirconium, lithanium, lead, and thallium,—and is not considered a metal proper. I say "not considered," although I myself fail to see why we chemists keep up a distinction of metals and non-metals, if we consider tin not a metal and osmium a metal. In a horizontal group, as chemists call it, it belongs to the series silver, cadmium, indium, tin, antimony, tellurium, and iodine. This group has still many absurdities. For instance, chromium belongs between sulphur and selenium, which is an equivalent absurdity; the manganese between chlorine and bromine; the cadmium between strontium and barium. But chemistry is full of historical reverence. At any rate, if tin combines with silver into a chemical amalgam, it can be in the ratios of $SnAg_4$ or $Sn_2Ag_2$, the first to contain 21.4 per cent. of tin and 78.6 per cent. of silver; the second 35.3 per cent. of tin and 64.7 per cent. of silver.

I have here these two atomic alloys, and you can judge of their quality. They do not seem to be much more than mixtures, approaching nearer silver the more they contain.

To consider the effect of mercury we may suppose the mercury distributes itself over the tin and silver according to their affinities. As we have seen from the native alloys, almost any range of mercury and silver is possible. Thirteen per cent. of mercury seems to be the lowest, and sixty-eight per cent. the highest; the highest that crystallizes, therefore the most likely to occur from an excess of mercury, is that with sixty eight per cent.

Tin forms a crystallizable compound with mercury, containing fifty-six per cent. up to seventy-eight per cent; hence it may be said that, in the average, the amalgam can take up and crystallize with seventy per cent. of mercury. In the process of mixing, squeezing, and working the amalgam, a number of stages are of importance. First, the grain of the tin-silver alloy. The coarser the grain the less mercury is taken up in the first instance; the more slowly the process of hardening, but the harder the amalgam, the less likelihood of shrinking. The finer the grain the more rapidly it hardens, takes up more mercury, and is less likely to expand than to shrink. This matter of fineness of grain seems to me of more importance than even slight variations in the composition. The difference of preparing the amalgams for a cavity by the various practitioners would more than account for the difference in the final result.

As to the various admixtures, I would say that personally I consider gold and platinum as worthless. In very small traces, and made at a high temperature, they may, similar to carbon in iron, harden the compound a little, but I should not expect any such effect with any large percentage, say above two per cent. The great trouble with amalgams in the mouth does not seem to be so much their lack of hardening,—they seem almost all to be hard enough,—but with their blackening, setting, shrinking, and working qualities. In regard to the shrinking of amalgams, it yet appears to me a disputed point whether the cavity may not shrink away from them. A tooth is supposed to be far more unyielding than it really is, and I have little doubt that teeth undergo slow changes of shape, whether dead or alive. The gentlemen here will probably know more about that than I; but from the fact that the slow changes of temperature alone would, in the course of some years, make a plug loose, even in metal, it seems to me very likely that such changes occur also in the mouth.

Considering the fact that we may have to drill through amalgams, cut and scrape them, any more hardness than is needed for the purposes of the mouth does not seem desirable. The subsequent changes and blackening are complex. I have little doubt that the condition of the mouth is a more important element than that of the amalgam.

How have the various devices worked,—for instance, lining the cavity previously not quite up to the margin with oxyphosphate, and then finishing with amalgam, leaving no oxyphosphate exposed to the action of the mouth, and allowing the amalgam to anchor essentially in the oxyphosphate? The lining with foil, tin, zinc, or aluminum,—how have they worked?

I should especially recommend aluminum foil for such purposes, because no compound of aluminum is black. The foil is made for the white signs under glass, and is quite cheap. It keeps the mercury entirely away from the cavity, because it does not amalgamate in the least.

Now, as to the other amalgams. By a relatively simple process amalgams of mercury with almost any other metal may be obtained, even with sodium or platinum,—namely, by using the mercury as the cathode in a solution of the metal. Of these amalgams, all those decomposing water may be disposed of at once; those are with the metals of the alkaline earth and with zinc; those which are very brittle or oxidize rapidly are also of no use. This throws out those of iron and the aluminum and platinum metals. Some good amalgams may be obtained with bismuth and cadmium. The first sulphurize easily, and the second—I do not know why—have a bad reputation among dentists, probably because the first amalgams, when dentists did not have the practice, were made with cadmium. So amalgams with titanium and wolfram and molybdanium might be useful, but the metals are terribly intractable: to melt molybdanium is just as much more difficult a job, compared with platinum, as the latter is compared with silver. Nickel and cobalt amalgams oxidize rapidly, thus showing that the nickel has its good qualities only when deposited in the solid sheet. Very good amalgams may be made from the remelting and renewing of old amalgams. A certain amount of mercury remains, even at a white heat of melting. This small amount causes subsequent mercury to be taken up much more readily, and as a consequence the amalgams harden with more rapidity; hence, where rapid hardening is desired, such old amalgams renewed have even certain points of advantage. As a practical matter, I would ask a little of the price of amalgams. With silver at eighty cents an ounce, and pure tin at about two cents, the price of the metal will vary between those two figures according to the proportion of each. Have you ever tried to file a large quantity of amalgam? It may be said that with a good file two ounces an hour is about all you care to make. I have tried, by casting the amalgam in proper shape, to work chips on the lathe, and I find those fine shavings of excellent quality, even superior to filings in some directions.

If you prepare the two atomic amalgams, you will find that, strange to say, the one with less silver is by far the whitest and hardest, amalgamates much easier, and hardens more rapidly. The one with more silver has a much darker natural surface, and, when filed and polished, a slightly yellowish tint, considerably darker than the one with more tin.

From these facts I would say that only the alloy $Sn_2Ag_4$ partakes of some of the elements of the chemical compound. Both alloys are fusible only at a much higher heat than would be the average of the melting-point of each constituent. The alloy $Sn_2Ag_4$ casts very well, while the one $SnAg_4$ is slightly pasty, even at a temperature of melting silver.

The chemical character of the compounds is of course not very marked, but sufficiently so to speak in favor of an alloy, $Sn_2Ag_4$. $SnAg_4$ does not take up the mercury very readily, and does not harden nearly as quickly as $Sn_2Ag_4$. The latter warms slightly.

DISCUSSION.

*Dr. Mayr.*—The amalgams used in dentistry consist of silver, copper, tin, gold, and mercury Those which occur in nature are certainly suitable as standards of stability, as for long ages they have been exposed to different influences, and have stood them all. There are numbers of amalgams occurring in California, Mexico, and South America. The mercury existing in them ranges from fifty-six per cent. to thirteen per cent.; we may therefore go as high as fifty-six per cent. without destroying a definite compound. The highest is well crystallized and very soft, and the lowest is almost like silver.

There exist also some gold-and-silver amalgams in nature. If you did not know their nature you would suppose that they were very valuable; they are found, however, to be entirely worthless, except for the gold to be extracted from them. A gold-and-silver amalgam which contains fifty-seven parts gold, thirty-eight parts silver, and five parts mercury, is absolutely powdery; that is to say, you can crumble it in your fingers, showing that the gold does not give very good qualities to amalgam. We frequently hear remarks similar to this: "What is there about gold that is of any intrinsic good? The color is like that of brass, and as for its weight it is of no use at all. For instance, build a bicycle of gold, and it would weigh a ton." But all superstition must be removed when we come to talk purely on a technical point.

The maker of amalgam would like to have a little gold emphasized in it, but I think for intrinsic value the gold is of very little use. Perhaps one-half of one per cent. or one per cent. of gold and platinum, when used in a high temperature, may improve the quality of the amalgam to a very slight degree; but they do it by their disappearance, not by their showing. The carbon which makes steel out of soft iron does not show in the steel; it has become a compound; so the gold is not there as an alloy, but as a slight chemical compound with the tin and silver.

I have made a number of experiments in regard to the tin and silver. As you know, tin chemically is a member of the carbon group, to which belong carbon, silicon, titanium, germanium, zirconium, tin, lithanium, lead, and thallium. In modern chemistry tin is not called a metal, but if we do not call it such all common distinction of metal or no metal disappears. We must consider tin like oxygen. The silver forms two oxides,—suboxide and monoxide.

Tin has four values. It can combine with four atoms of different univalent elements. I can have four atoms of silver combine with one of tin, or, by double combination, four atoms of silver with two of tin. Those are the two chemical compounds between silver and tin. Now, which really exists? I made the compounds, and I find that the tin which should be $SnAg_4$ does not oxidize; but there exists a compound of two of tin and four of silver. That is the compound which is the analogous compound of tetrachloride of tin. When I fused tin and silver perfectly pure, I found that the compound which has the most silver is entirely worthless for dental purposes. It is much darker than the one with less tin; it amalgamates very slowly, hardens poorly, is rather soft, and has the qualities of a mixture of tin and silver. The first amalgam, which I have here, contains seventy-eight per cent. of silver and twenty-one per cent. of tin. This high grade silver amalgam is of no value at all. You could not tell whether the mixture was silver or tin. The identities of both metals have been lost; therefore it partakes of the characteristics of a chemical compound. I have both specimens here. This second compound also is very hard—much harder than silver or tin alone. It is whiter than tin, and has a different cast from silver. It is very difficult to drill. The amalgam which you make from it hardens very quickly. It was made this afternoon before I left my home in Springfield, and it mixed very nicely. I find that dental amalgams should be of the formula of sixty-four per cent. of silver and thirty-three per cent. of tin,—that is, about two parts silver and one part tin. The other formula of three and one-half parts silver to one part tin is of no value. Here is the amalgam which I spoke of. It has not hardened yet. The color of the first, with the higher percentage of silver, you will notice, is a little brighter and more shiny, but is much darker. You could readily tell which compound contained the more silver. This amalgam is still soft, while the other one, with less silver in it, is perfectly hard and strong.

Now we come to the point of the discoloration of amalgams. That cannot be prevented in any amalgam of tin and silver. The natural amalgams are not superficially dark, showing that it is not a natural atmospheric influence that causes it. It is due, in my opinion, to some sulphurizing influence in the mouth. The natural amalgams, which are constantly exposed to moisture and oxygen, do not darken. If you will put any of those amalgams in a solution which oxidizes very easily, you will find them to retain their color; but put the least trace of sulphur in, and they immediately turn black.

By lining the cavity with aluminum-foil I think you could prevent the darkness of the amalgam from showing. Aluminum has the great advantage of not alloying with mercury, but I have never heard that it has been used in dentistry. It makes an excellent lining and a perfect protection against anything showing through.

I heard from your minutes this evening that Dr. Lord gave a short paper on amalgams combined with oxyphosphate at the last meeting. I think if the oxyphosphate is kept from the very edge of the amalgam, that it will stand as long as any one desires, and at the same time will prevent the darkness from shining through.

*Dr. Hodson.*—About the aluminum-foil: the foil I have seen has been very harsh, and would scarcely mould to the inside of a cavity, and if we should wish to use it, there is no certainty of not splitting it before we get through. Can it be softened?

*Dr. Mayr.*—It comes in quite thin form. I have received it several times as thin as tin-foil. That which is ordinarily used for window signs is fairly thick, but I have seen it imported from France so thin that it could be easily folded. It is proof against mercury. It may dissolve and may oxidize, but it forms a compound which will not let the amalgam shine through. If you were to try to anneal the aluminum-foil, it would not crumble if you put it in a glass vessel and put in a test-tube into which hydrogen gas can be introduced. I think that you will find that it could be annealed very easily.

*Dr. Hodson.*—I have tried it, but it was so hard I could not get much comfort from it.

*Dr. Chester.*—The subject of amalgams is not one that I have made any study of, except in a general way. I have perhaps studied the use of gold more than of any other metal. I have been interested in Dr. Mayr's paper and the discussion of it. It has opened a new field of thought on the chemical side. I can hardly agree fully with the essayist as to the worthlessness of gold. I cannot agree with him as to its lack of beauty. I think it is a very beautiful metal, and am sure we all like to see it.

There are some curious things with reference to the combinations of metals with each other. We speak of amalgams and alloys, and it is somewhat a question as to just what is meant by these terms. An amalgam is a combination of a metal or metals with mercury; to have amalgam, then, mercury must be present. Other compounds of metals are known as alloys. If they possess the characteristics of a new substance, as the alloy of silver and mercury, or amalgam, as we may more properly call it, that has just been described, there is no reason why we may not consider it a chemical combination rather than a solution of the metal in the mercury, and it seems to me that this view is in the line of later chemical thought.

I wish to ask Professor Mayr if he has found any crystals in any of his experiments with the particular amalgam of which he spoke?

*Dr. Mayr.*—Only in the tin-and-mercury amalgam which is used on mirrors. Certain crystallization can be seen, although not clearly defined. You can occasionally see little facets under the microscope. That is a compound which varies in mercury. I have found from fifty to seventy per cent. of mercury on the back of mirrors.

*Dr. Chester.*—This question of crystallization was one of the first that came up when it was sought to prepare, thirty or forty years ago, a crystallized gold, which afterwards came into the market as sponge gold. In 1850, Dr. Watts, who afterwards brought out gold in the fern-leaf form, began to make preparations of sponge gold, and in 1853 he obtained the first of these. It is interesting to know that the discovery of it was the result of careful experimenting on the subject, and not at all accidental. That material was made first by dissolving purified gold,—gold made as pure as it could be by precipitation with copperas,—and washing, and then the finely-powdered material that was left was dissolved in mercury and heated for some hours to not a high temperature (I do not recall just the figure, although I have often gone through the process) until there seemed to be a combination between the gold and the mercury which differed from a simple amalgam. Then the material was dissolved in nitric acid, which will take out a large proportion of the mercury, and a mass of crystals was left, containing about six per cent. of mercury, the rest being gold. This seems to be a definite amalgam of gold and mercury. The crystals are always the same in shape, no matter how many times you prepare it. I presume many of you have seen them under the microscope. You have then very small crystals of nearly pure gold, containing a little mercury; this, when afterwards subjected to heat and annealed, forms into a spongy mass, which can be worked as a sponge gold in filling teeth. When this was discovered, it was a great step in advance. It was brought before the profession in 1853.

It seems to me clear that such amalgams, as they are called, are definite chemical compounds, with a definite crystalline form. The native amalgams of silver all crystallize in the isometric system. The only one of gold that was ever found crystalized is also in that system. I have received crystals of gold taken from works where mercury was used in amalgamating gold which were also of the isometric system, but these artificial crystals are always similar, and belong to the hexagonal systems, and I believe them to be an amalgam of ninety-three or ninety-four per cent. of gold, with six or seven per cent. of mercury.

The combination between the gold and mercury seems to require some time and, perhaps, heat, as in the case mentioned; unless the operation is conducted in this way, and the materials heated together for a certain length of time, the residue, after driving off the mercury, is a coherent mass of gold, with none of the characteristics that are desired. The use of mercury for the production of an amalgam which shall be valuable seems to me to depend on a chemical combination. That metals may become combined chemically so as to produce results which formerly would have been considered impossible has recently been proved. I have an alloy of gold, silver, and copper, in the proportion of about sixty of gold, or a little more, a little more than thirty of silver, and about five of copper, which, though containing those proportions of the metals, crystallizes perfectly. Formerly we considered it impossible for such a thing to take place.

There is one thing that needs a great deal of investigation with reference to this material for filling teeth, and that is, how much there is of chemical combination and whether the perfect material, or as nearly perfect as possible, is not one where a chemical combination takes place after the material has been put into the teeth; and therefore takes place in such a way as to produce a much more permanent result than if it were allowed to combine chemically first, and then put in. It is like the setting of plaster of Paris, which is really a chemical operation. If it once sets, unless in the place where it is wanted, it is of no further use.

How can we mix materials so that they shall have the property of working under the instrument properly, and yet of setting quickly, so they may produce their chemical combination just while we are at work on them, and get through with their shrinkage before we leave them? I do not know that I can suggest how it may be done, but there is a grand opportunity for investigation in this line.

*Dr. Mayr.*—Shrinkage has been mentioned. I have always been an upholder of the opinion that changes occur in teeth, and it has been my opinion that the tooth shrinks away from the amalgam, and not always the amalgam from the tooth. We consider the tooth altogether too immovable and non-changing. The teeth undergo slow and steady changes. Take, for instance, a piece of copper, drill it carefully, set in it a plug of gold that is not tapered too fine, and let it stand for a while. That plug of gold is loose in a year, simply from changes of temperature. So I think with the tooth. The contraction and expansion of tooth-structure and amalgam are extremely different. When you drink cold water, the amalgam responds at once and the tooth very slowly. The amalgam, for instance, appears sometimes to have swelled out above the edges of the tooth, when, in my opinion, it has been crowded up from underneath by the frequent changes of the tooth-structure. I think that often explains cases which we cannot otherwise understand. From making experiments in glass tubes, I do not think very much shrinkage takes place in amalgams. The elasticity of a tooth would always be up to the requirements, so I think the shrinkage of the material cannot be decided.

The next point is, in what shape we want our alloys for working into the tooth. Do we want powder, do we want grains and shavings? I think sometimes the difference in two makes of amalgam is due to nothing but the difference in grain. If you take an amalgam with a high percentage of tin, and drill into it, you will find that you get a powder,—you do not get chips. That powder hardens rapidly, and contains more mercury in proportion than the other amalgam with a higher percentage of silver. With the other you get little chips, which take up the mercury very slowly. Apparently it does not harden, but remains crumbly a long time.

The question of grain ought to be viewed carefully. If we want a quick-setting amalgam we must have a fine powder. A slow-setting amalgam, and one that takes up the mercury thoroughly, is a coarse grain. One good plan I found was to cast the alloy in a shape for the lathe and then turn off a small chip. That gives a mixture of fine but essentially small shavings. I find those chips and shavings take up the mercury very nicely, and more regularly than the powder. I think that point explains the difference in many kinds of amalgam. I am in doubt whether the gold in amalgams does any more than temper it a little. Suppose we had cooking-vessels of gold. Every housewife would groan at the weight of those vessels, because gold is so soft that the vessels would have to be made an eighth of an inch thick. Copper is certainly far preferable for that purpose. Suppose we had gold wire instead of the ordinary wire we use. It would break from its own weight. Water-pipes could not be made of gold. What could we use gold for, if not for money? As to the aluminum, it is one of the industrial fakes until its price falls below that of zinc. I have in eight years not been able to find a use in my laboratory for anything made of aluminum. Sometimes I could use it by fusing it with other metals, but by itself I could do nothing with it. So with gold. You cannot drill it nicely, and cannot do anything with it. As to its beauty, that is a matter of taste. I think gold looks like brass, and I see no beauty in it. The intrinsic value of gold is not great. As for filling teeth, "there is not enough gold in the United States to fill the teeth of all the old women," as some one in Kansas said recently; at the same time, gold fillings have a great advantage in many cases, in that the finish does not change. Gold in plates has great advantages over platinum, and certainly over aluminum plates. Dr. Searle, of Springfield, had aluminum plates about forty years ago. He found that after very little use the food and acids ate little holes in them. I would like to know how those plates have worked of late. They have been introduced again, and I think with better success.

*Dr. Watkins.*—Professor Mayr said that discolorations of amalgam fillings were due to sulphur and moisture. I would ask him if that discoloration is not due to the moisture becoming incorporated in the amalgam before it is set or thoroughly packed in the cavity? Several years ago I made a number of experiments in filling cavities with different kinds of amalgam, for the purpose of testing and deciding upon a certain kind for my own use. To illustrate I will describe one case which I think will explain the matter thoroughly. I had a young lady with extremely soft teeth. There were large cavities on the grinding and buccal surfaces of each of the molars. I placed the rubber dam on those teeth; then filled the cavities with different kinds of amalgam,—for instance, using Lawrence's on the grinding surface of the wisdom-tooth, Dorsen's on the buccal surface of the same tooth, Arrington's on the grinding surface of the second molar, Dibble's white alloy on the next one, and so on, with the idea of watching those cases and discovering which retained the best strength and the best color. In the case to which I have reference, I put in some seven or eight kinds of amalgam. I have seen that patient about once a year ever since for the past eight years, and can see no appreciable difference in the color of all those different kinds of amalgam. The Lawrence amalgam, which is generally supposed to turn very black, is just as light as the Dorsen amalgam; not exactly the same shade; perhaps a little more of a steel-color than the Dorsen, the Dibble, Shumway's, Hood & Reynolds's, or Welch's; but the amalgams which we have always supposed to turn dark immediately were just as good a color as the others, so there was no appreciable difference. So far as the edge-strength was concerned there was no comparison. The Lawrence amalgam had the best edges of any. I do not know what the proportion of silver is in that amalgam.

*Dr. Mayr.*—Is that not a high silver amalgam?

*Dr. Watkins.*—I do not know. It is very hard when I pack it in the cavities.

*Dr. Lord.*—Professor Mayr, as I understand him, thinks that it is a question whether the amalgam shrinks, or whether the tooth shrinks away from the filling.

It would seem that his mind could be readily set at rest on that point, as it is not possible to suppose that there can be any shrinkage in the tooth-structure.

There is often found to be a space between the amalgam filling and the walls of the cavity, but it cannot be owing to any shrinkage of the latter, and can very easily be accounted for on other and positive grounds.

There is an appreciable shrinkage of amalgams made of an alloy of two or more metals. This is generally understood, and it constitutes the great objection to the use of amalgam, though it may no doubt be employed as a filling where it is specially called for, with advantage over other materials.

If Professor Mayr can suggest an amalgam that will not shrink, or can make one, he will confer a very great benefit on mankind.

*Dr. Mayr.*—The whole reasoning proceeds from a supposition that the natural condition of the setting of the amalgam would be shrinkage. In putting an amalgam in glass you cannot discover the shrinkage. Let it stand a year, and it is loose. What has caused it? Not the shrinking of the amalgam, but the change of temperature. So with a certain degree of temperature the amalgam was compressed by the glass, and, not being totally unyielding, it went back a little more than it had been at first. That slowly loosened the amalgam in the glass. When it was first made, it could not be moved at all.

Have not many of the gentlemen put in gold fillings in rather doubtful teeth? You have made it so the gold would not move. Suppose you had made a nice job, and the person came back to you and said, "I have lost that gold filling." What would you think? The gold did not shrink; the tooth has changed enough to loosen the gold. I have seen such a case in a large gold filling made by an experienced practitioner. A lady came to me and said something odd had happened. She was drinking coffee, and bit on something hard, and there was a piece of gold in her mouth. It had come out of her tooth. Have you never observed this, especially in dead teeth? If you take a substance like a tooth, with a cavity inside, and make it shrink in its substance, the cavity does not grow smaller. The wall shrinks from both sides. That makes the cavity larger, although the tooth has shrunk.

If you make a cavity of glue, put in a filling, and let the glue dry, how does your filling behave? The gold does not shrink, but the soft substance moves away from the cavity on the one side, and from the air on the other. I think that process goes on more than we give it credit for. As soon as the tooth gets nourishment, we have changes in shape. When a dead tooth grinds against a healthy tooth, the dead tooth wears disproportionately. The healthy tooth gets nourishment from beneath, and the dead tooth does not. In order to find changes, we would have to photograph the molars of a child, and then photograph the same tooth forty or fifty years afterwards, when I think you would find that the tooth had materially changed in shape. Of course, a few years of observation would not show.

*Dr. Lord.*—Professor Mayr thinks that the temperature would affect the changes in an amalgam. Is the change of temperature in the mouth, under the influence of the regular temperature of the body, sufficient to cause any expansion or contraction?

*Dr. Mayr.*—That change could come only from the food. If you eat cold dishes or warm dishes, the amalgam will respond to them. There is constant contraction and expansion going on, which slowly loosens an amalgam in a doubtful cavity, and a gold filling in the same way. I think the case I mentioned was caused in that way. The lady masticated on that side, and probably got all the hot and cold food there.

*Dr. Chester.*—I think it is the experience of most of us that we do not let ice-cream and ice-water come against our big amalgam fillings when we are eating, if we can help it. Dr. Mayr is certainly correct in reference to the popular idea being a wrong one, that there is necessarily shrinkage when metals are combined. We have found that out in making casts from various kinds of metals. When they are cooled from the liquid to the solid state, which is comparable with the change that takes place when an amalgam sets, some expand and some do not. If it is true, as is generally believed, that there is a shrinkage of amalgams, there is at that point a chance for examination and investigation to produce an amalgam which will not shrink, or which will keep its shape exactly after it has once set.

I want to say a word more about gold. For certain purposes it seems to me that gold is the ideal material for filling teeth, because it is absolutely insoluble in the fluids of the mouth. It is not changed chemically, and it is a pure substance. We do not have to consider what the alloy should be. Of course, the gold that is used in filling teeth in general is not quite pure gold. Cohesive gold is as near pure as it can be made. Gold should be made pure at first, and then copper put with it to give the proper consistence. One grain in four ounces would only be about one two-thousandth part, yet that has a very decided effect, changing it from cohesive to soft foil. The fact that it is a pure material, unacted upon by any chemical substance which would probably get into the mouth, makes it an ideal substance. If it could be worked as amalgam can, I think it would be used altogether, because the difference in the cost would not be very great on any particular filling.

I have supposed that if sponge gold could be made that was pure and would never discolor, it would be of the greatest value for certain parts of dental work, and I know that such a material can be prepared. I believe that the discoloration is not due to any impurity in the gold, but to a lack of care in the treatment of the material. It will not do to wash the gold with ordinary Croton water; it should be washed with distilled water to make it pure, even though it were to be melted down afterwards. I think its discoloration is one reason why many dentists have discarded sponge gold, simply because it was not properly prepared. Foils have been through the fire, but sponge golds have to be taken out of water, or some other liquid, and then dried, and, though they are annealed after that, there is not the same opportunity for purification. They can be purified, but it is not always done, as many of you have learned.

*Dr. Northrop.*—Professor Mayr's theory is one that I do not believe, and do not want to believe. It would knock the foundation out of honest dentistry. If Professor Mayr can demonstrate to us the true measurement of the cavity and the true measurement of the filling, and show us that that cavity has shrunk or enlarged from the filling, we should be obliged to believe it. Seeing might, under those circumstances, be believing; but hardly then. If a tooth has undercuts and still the cavity enlarges, it must admit moisture. The tooth would disintegrate and the filling be of no use whatever. If Dr. Mayr's theory is correct, operative dentistry is a farce. I should like to have him give us a demonstration of that theory.

*Dr. Bogue.*—I once took the trouble, with Dr. Lord's help, to get an instrument which would measure the movements of amalgam to about the thirty-two one-thousandths of an inch, and that measurement was magnified with the aid of a long needle; and every amalgam that has been mentioned here to-night, and a great many more, were examined. That little instrument was carefully watched; the room was kept at a uniform temperature day after day and night after night. Two amalgams did not shrink, but every other one did, and expanded, too. In the act of congelation, precisely as zinc and iron do, those amalgams expanded and then contracted.

In regard to the necessary behavior of amalgams, let any gentleman present imagine either a drop of lead melted, or a drop of mercury. Imagine that drop put upon a plate hot enough to keep it melted. Put into that drop filings of any metal for which that melted mass has an affinity. Let it be mercury, by way of argumentation. Put into it the filings of any amalgam you choose, only so the affinity be present. Your mercury will flatten. Put in some more, and it will flatten more. By-and-by it will assume the shape of a little cheese. Continue your addition of filings of alloy to the last possible degree, and you can never make square corners; here is where Dr. J. Smith Dodge should receive our highest compliments, for he, as far as I know, was the first man to announce a spheroidal tendency on the part of amalgams.

There are two metals which do not contract: they are copper and palladium. They will make a sharp, square edge, and my instrument, which measured very finely, did not show any contraction on the part of either. On the part of the others, there was contraction, evidenced in some of them up to eight or ten days after solidification had taken place, and there was that tendency to a spherical shape in all of them. If Dr. Mayr can get up an hypothesis, I can too. We may assume that this tendency towards a spheroid is of universal existence. Perhaps he may say I cannot prove it. Well, I cannot; but I think this hypothesis is as good as any he has brought up to-night.

There are questions that come up to which, perhaps, it may not be necessary to allude. I do not like to see ideas emanating from this Society which are so manifestly incorrect.

*Dr. S. G. Perry.*—We all know that a drop of water takes a spherical shape. It may be true that amalgam fillings have the same tendency. We know that large fillings shrink and fail more readily than small ones, and this might be expected if this hypothesis is true. But there is another important factor which should be considered. Like the ivory of billiard-balls, the teeth are more or less elastic. Amalgams as well as cohesive gold fillings are inelastic, and the inharmony between the tooth and the filling is doubtless partly responsible for the bulging out and final failure of many of those large fillings. In saying that teeth shrink, I think Professor Mayr was unfortunate in his phrase. They crumble away from the fillings, and have the appearance of shrinking, but I cannot understand how this can be possible. The soft gold fillings of the old operators were more elastic and more in harmony with the teeth than the modern ones that are made of cohesive gold and are hard and inelastic.

*Dr. Bogue.*—I am sorry Dr. Perry did not speak of another thing which he knows exceedingly well; that is, the checking of the margins of large cavities when fillings are inserted.

*Dr. Perry.*—That is so important a factor that the time has gone by for the making of enormously big gold fillings of strictly hard cohesive gold.

*Dr. Bogue.*—Both Dr. Perry and myself have been experimenting with amalgams since those memorable experiences in 1873. Last week one of my regular patients was in my chair. When he was a boy of ten or eleven I put in amalgam fillings, some of them quite large, in the front lower incisors. Those amalgam fillings have been there a long time, I expecting every year to exchange them and put in gold. The time has not yet come for me to do so. What is the reason that one amalgam filling is a good one and another is not? There were fillings put in when I knew nothing about amalgams.

*Dr. Jarvie.*—Have those fillings oxidized?

*Dr. Bogue.*—Not very seriously. They are in the lower incisors, on the approximal and anterior surfaces. Parts of them are accessible to the brush, and parts are not, but are accessible to the silk which he uses.

*Dr. Northrop.*—Did you apply the rubber dam?

*Dr. Bogue.*—That I do not remember. There was an allusion by Professor Mayr to-night to the use of such materials as should utilize chemical affinity. I do not know whether it is generally known that silver and mercury have a strong affinity, and that silver and mercury in proper proportions will set with wonderful rapidity. I spent two days last summer in London, for no other reason than to investigate palladium, and I have not had such an interesting hour as I passed with Mr. Matthei. He has written a fine treatise on palladium. Mr. Matthei is to-day probably the owner of all the palladium that is known to exist in the world. It was found in a gold mine and was thrown out as refuse. He picked up some of it, worked it out, and found various qualities in it that he considered good. Mr. Rogers came along and wanted to see if he could not utilize it in dentistry. Plates were made of it. From plates it got to fillings, and Mr. Matthei got out a lot of gray precipitate, which was used for some time. To show how uncertain that matter is (I have had quite a correspondence with him since), he provided me with about half an ounce of palladium when I left there; the affinity of mercury with this lot was so great that it actually exploded whenever I used it. Then he sent me a larger quantity, perhaps an ounce and a half. With it he sent me a bit of palladium already amalgamated in a glass tube sealed at both ends. This amalgam, when it reached me in Paris, was soft. When I got to New York it was still not very hard. The affinity was not so great. He wrote me that he understood that palladium could be and had been used in the same fashion as copper,—that is, it was mixed up in the morning, and the mercury squeezed out when it was wanted during the day. The way in which Mr. Matthei discovered the wonderful affinity was that he was rubbing it in his fingers together with mercury one day, and he squeezed out so much mercury that it dripped along the floor; finally he got out so much that it exploded and burnt his fingers. I have made a good many palladium fillings, but I carefully weigh out my mercury and my palladium and mix them quickly. The fillings set nearly as soon as I get them in. The whole subject of amalgams lies open for some one who has the time, means, and patience to investigate it. I sent a lot of amalgam scraps to Professor Mayr some time ago to melt up and get into fillings again, in which I have found some very curious qualities.

*Dr. Mayr.*—It was the most curious mixture I ever got hold of. It was very hard to get rid of the mercury in the first lot.

*Dr. Bogue.*—And yet it was the nicest amalgam to work that I ever saw; it mixed smoothly, set very quickly, had good edges, and remained white in the mouth.

*Dr. Jarvie.*—What has been the result in the teeth?

*Dr. Bogue.*—I cannot say, because it is only about three years that I have used it.

*Dr. Jarvie.*—If a filling remains in a tooth perfectly intact for three years it is pretty good testimony.

*Dr. Bogue.*—The fillings have served thus far admirably. They are made of some amalgam scraps of which I do not know the exact composition, although it has been analyzed since it was last melted, and traces of mercury were found in it. It seems as though its former admixtures have contributed to its present properties.

*Dr. Perry.*—Are you using palladium still?

*Dr. Bogue.*—Yes, when I can get any that is good. What I have now does not amalgamate readily.

*Dr. Hodson.*—What was the composition of the scraps you sent to Professor Mayr?

*Dr. Bogue.*—A lot of old amalgam scraps.

*Dr. Hodson.*—Was there any gold among them?

*Dr. Bogue.*—Yes; I generally wipe off the surface of my amalgam with gold.

*Dr. Mayr.*—You refer to a lot of old amalgam scraps which were fused down?

*Dr. Bogue.*—That amalgam had been melted down by Professor Mayr and filed down. There was Dibble's amalgam in it, Shattuck's, Fletcher's plastic, and some others. There was a great deal of gold. In putting in my amalgams, the first pieces are fairly soft; after that just as hard as I can get it; then they are packed into place and rubbed with tape to make it assume the shape of the tooth. Scraps of that sort I sent to Professor Mayr, and the influence of that mercury and that former mixing, it seems to me, are the only things that can explain the curious results that were attained.

*Dr. B. C. Nash.*—Is it not a fact that the most satisfactory amalgam fillings that we see in the mouth are those that are absolutely black, whether they be of copper, whether they be regarded speculatively as Lawrence's amalgam, or those amalgam fillings in contact with gold fillings, which I have frequently observed of inky blackness? Do we not generally find that such fillings have not shrunk; that their edges are clean, and that the tooth-structure is not discolored?

*Dr. Mayr.*—I am extremely glad to hear in regard to this palladium amalgam. I find Dr. Bogue got them by using mercury in a strong solution of palladium. I worked it up, and then I had to free that little lot of palladium from the mercury, and work it over, and I became highly disgusted. Finally I melted it all together, and so at least Dr. Bogue did not lose anything.

The subject has been referred to, that I at one time stated a tooth would grow enamel again. That was not said in the sense in which it was taken. I was drawing from analogies, and was speaking of Dr. Heitzmann's observations, where we can see newly-formed dentine within the tooth, in response to reparative work. Would it not be possible that to a certain degree enamel can be supplied, of course, in a very moderate degree? One doctor told me he observed a cavity that was one-fourth of an inch from the edge of the tooth; now it is only one-eighth of an inch from the edge of the tooth, yet the tooth is as long as it was. Did that grow dentine from the root, or did enamel move from the dentine of the tooth? The tooth is as long as it was before.

I like an hypothesis as a guide. Life is too barren to live without them. We make hypotheses about everything, otherwise we do not get any new ideas.

About shrinkage,—a dry tooth is decidedly shrunk. I have seen that. When you dry the cavity you dry the tissue. Dr. Northrop's test is too hard. He says, let us take a cavity and measure it, and see if it changes. I could not have a patient walking around with a little measuring apparatus in his mouth, seeing where the cavity changes. The test is too difficult. Dr. Bogue has probably made the experiment of putting the amalgam in glass. Does the average amalgam allow any bacteria to go through between the glass and the amalgam? Do any microbes go through? The difference must be the size of the smallest microbe. The size of a microbe is one twenty-five-thousandth of an inch. That the opening does not go beyond the one twenty-five-thousandth of an inch, I think, is proved. Absolute contact does not exist in anything. If, for instance, gold and silver are rolled together and begin to adhere, then we have absolute contact, but otherwise absolute contact is not known. The varnish that sticks to wood does not thoroughly adhere to the wood. There is air everywhere between them. So, actually, I do not think we get thorough adhesion. If you dry the cavity and put the amalgam in, then the natural condition of the tooth coming on will again take up its natural moisture. So to a slight measure you get from a tooth, assuming its natural condition, a swelling. There is nothing very improbable about that. We do not have feet and inches to measure by; we have ten-thousandths of an inch,—very small measurements.

The Secretary then read a letter from Dr. Hathaway in regard to the amalgam question, an abstract of which is as follows:

". . . A few years ago I attempted to do something in the line of experiment, noting the results of various combinations, but my notes having been by accident destroyed, I cannot now speak except in a very general way.

"I was, however, impressed by the widely different results obtained from alloys made as nearly as possible under the same conditions, and varying in composition only by small percentages,—in some cases only one-half of one per cent. difference in the amount of gold or zinc. . . .

"My process is simply this: With a well-ignited coal-fire in my laboratory stove,—or, better, with a charcoal-fire,—the crucible, first rubbed over the inside with borax, is gently heated and the tin melted first, then the silver is added in small pieces, and the melting mass, which is kept at as low a temperature as possible, is stirred with a clay pipe-stem or an oak stick. As soon as the silver is melted, the gold, or copper, whichever is used, is added,—the copper in the form of wire rolled as thin as possible. As soon as the melting is complete it is quickly turned into a cold ingot mould; then all that remains is to file with a somewhat coarse file, and remove bits of steel with a magnet, and the alloy is ready for use.

"Two formulas, which I make constant use of, are given,—viz.:

| | | | |
|---|---|---|---|
| Silver | 55 parts. | Silver | 45 parts. |
| Tin | 40 " | Tin | 45 " |
| Copper | 5 " | Gold | 10 " |

"One gives a black amalgam, the other a light-gray; both have good edge-strength, shrink but little, and take a good polish. I have made many others, but these are my main reliance.

"The four metals named are the only ones I consider of value. Platinum seems to me to be nearly or quite inert, and zinc, while it gives whiteness, gives also a greasy feel which I detest, and I suspect it tends to cause weak edges and the spherical form in crystallization. Of the latter I am not definitely certain, but do not now use it in my alloys.

"The preparation of the amalgam for the cavity is, I think, best made in the following manner, suggested first, I believe, by Dr. Rollins: The powder is placed in the mortar with a small quantity of a one- or two-per-cent. solution of sulphuric acid, the requisite amount of mercury added, and the mass rubbed up before the acid is poured off; the pellet afterwards washed in clean water. I do not believe in working amalgam as dry as some advocate. I want it plastic enough to pack well, and like to have it packed before crystallization has far advanced.

"The quality of mercury used is, I think, a matter of consequence, and may explain some of the difficulties with amalgam."

CASUAL COMMUNICATIONS.

The Secretary read the following communication from Dr. Crouse, with reference to the resolution offered by Dr. Littig at the last meeting:

"CHICAGO, June 16, 1893.

"DR. GEO. A. WILSON, *Corresponding Secretary New York Odontological Society:*

"DEAR DOCTOR,—Your communication from the New York Odontological Society is before me. In reply would say, if it is desired by the different dental societies that the Dental Protective Association take up the work of petitioning Congress to have the tariff or duty reduced on dental goods imported to this country, the Association will undertake the task. We believe, however, before such work is entered into we should have a distinct understanding with those importing goods to this country, as to whether or not the benefit of such reduction as might be made by Congress would go to those practising dentistry or to the dealers. This is a feature of the work which can be looked into and arranged with the importers, I presume.

"It seems to me that it would be wise for some one to correspond with the different societies, and, if they so desire, ask them to make the same request of the Protective Association before the work is undertaken.

"Thanking the Society for its confidence, I remain,

"Very truly yours,

"J. N. Crouse,

"*Chairman.*"

*Dr. Littig.*—I would move to lay this communication on the table until the fall meeting.

Seconded. Carried.

*Dr. Howe.*—I received a letter from Dr. Crouse yesterday, in which he sent me some burrs and some samples of amalgam that the Dental Protective Association are about ready to issue to the members. I have tried the burrs pretty severely, and I find them, in quality of cutting-edge and temper, to be very fine indeed; equal, I think, to anything I have ever used. Members of the Dental Protective Association will soon have an opportunity, I understand, to obtain these products at the lowest possible prices.

*Dr. Bogue.*—I received the other day a letter from Paris, containing an account of the most audacious operation in oral surgery that I ever heard of. The history of the case is very quickly given. A young girl had a resection of the lower jaw, from which was taken about two inches. Dr. Michaels was called in by Dr. Paion, the surgeon in the case. After the operation had been performed and the wound healed up, the cicatrix was reopened, the two ends of bone separated to their proper positions as they were previous to the operation, and a ladder of gold was put in and screwed to the two pieces of the lower jaw. The soft parts were put together and closed with a suture. The first operation was performed on February 4, 1893, and this letter of June 8, five months after, shows that the wound has entirely healed up, except in one small point, and the girl is quite comfortable. There is a photograph here illustrating it. Two inches of the lower jaw were taken out, one end of the gold ladder was screwed to the small piece at the ramus, and the other end to the larger piece towards the chin, and the whole apparatus was covered up by the integuments.

*Dr. Ives.*—I have a case of some interest to present, the result of an implantation; interesting as an operation, because performed by the apostle of the principle, and of late somewhat severely condemned by the prophets; performed in his own office in California, and presumably, therefore, under the most favorable circumstances. The lady, a patient of mine, came directly to me on her arrival here, and I gave the case the most careful attention. For some little time it did not look promising, but eventually seemed an assured success. I have not the exact dates to give you to-night, but I think it is a little less than two years since it was implanted. Six months ago it was perfectly firm and promised well. Two weeks ago it dropped out. It tells its own story better than I can. I can only repeat a remark I overheard Professor Heitzmann make at one of our meetings at the time Dr. Younger was presenting his method in this city,—"It may remain firm for a time, but it is mighty poor surgery."

*Dr. Perry.*—I do not consider that an argument against implantation. I have implanted nearly a hundred teeth since this method was first introduced. I do not suppose I have saved half of them, but I have saved pretty nearly half, and many of them have been in six and seven years, and they are considered priceless by the patients. Because a tooth has become absorbed I do not consider it an argument against implantation, for another can be placed in, and even two or three, if necessary. I have implanted three times in the same socket, and finally got a good result. Implantation has a place in dental practice, and should not be rashly adopted or quickly condemned.

Adjournment.

John I. Hart, D.D.S.,
*Editor New York Odontological Society.*

